# Comparative evaluation and analysis of DNA N4-methylcytosine methylation sites using deep learning

**DOI:** 10.3389/fgene.2023.1254827

**Published:** 2023-08-21

**Authors:** Hong Ju, Jie Bai, Jing Jiang, Yusheng Che, Xin Chen

**Affiliations:** ^1^ Heilongjiang Agricultural Engineering Vocational College, Harbin, China; ^2^ Engineering Research Center of Integration and Application of Digital Learning Technology, Ministry of Education, Hangzhou, China; ^3^ Beidahuang Industry Group General Hospital, Harbin, China; ^4^ Department of Neurosurgical Laboratory, The First Affiliated Hospital of Harbin Medical University, Harbin, China

**Keywords:** 4mC DNA methylation, deep learning, classification, feature, visualization, interpretable ability

## Abstract

DNA N4-methylcytosine (4mC) is significantly involved in biological processes, such as DNA expression, repair, and replication. Therefore, accurate prediction methods are urgently needed. Deep learning methods have transformed applications that previously require sequencing expertise into engineering challenges that do not require expertise to solve. Here, we compare a variety of state-of-the-art deep learning models on six benchmark datasets to evaluate their performance in 4mC methylation site detection. We visualize the statistical analysis of the datasets and the performance of different deep-learning models. We conclude that deep learning can greatly expand the potential of methylation site prediction.

## Introduction

The rapid progress in genome sequencing technologies has facilitated the investigation of the functional effects of DNA chemical modifications with unprecedented precision (Larranaga et al., 2006; Jiao and Du, 2016; Hamdy et al., 2022). DNA methylation, as a vital epigenetic modification, plays a crucial role in normal organism development and essential biological processes (Lv et al., 2021). In the genomes of both prokaryotic and eukaryotic organisms, the most prevalent kinds of DNA methylation include N6-methyladenine (6mA) (Huang et al., 2020; Li et al., 2021; Chen et al., 2022), C5-methylcytosine (5mC) (Cao et al., 2022), and N4-methylcytosine (4mC) (Moore et al., 2013; Plongthongkum et al., 2014; Ao et al., 2022a; Zulfiqar et al., 2022a; Zulfiqar et al., 2022b). The distribution of 4mC sites in the genome is highly significant as they play a crucial role in regulating gene expression and maintaining genome stability. Accurate identification and analysis of 4mC sites allow for a deeper understanding of the role of DNA methylation in gene regulation and disease mechanisms. This has important implications for the study of epigenetics, cancer etiology, biological evolution, and potential therapeutic strategies. Therefore, the development of efficient and accurate methods for detecting and identifying 4mC sites is of great importance for understanding biological processes and disease research (Razin and Cedar, 1991; Kulis and Esteller, 2010).

Several experimental techniques have been utilized to identify epigenetic 4mC sites. These methodologies include methylation-specific PCR, mass spectrometry, 4mC-Tet-assisted bisulfite-sequencing (4mCTABseq), whole-genome bisulfite sequencing, nanopore sequencing, and single-molecule real-time (SMRT) sequencing (Buryanov and Shevchuk, 2005; Laird, 2010; Chen et al., 2016; Chen et al., 2017; Ni et al., 2019). These experiment-based methods suffer from limitations such as low throughput, high cost, and restricted detection sensitivity. Nowadays, machine learning has been widely utilized and are successful technology in bioinformatics for extracting knowledge from huge data (Larranaga et al., 2006; Dwyer et al., 2018; Hu et al., 2020; Hu et al., 2021; Hu et al., 2022a; Zeng et al., 2022a; Zeng et al., 2022b; Li et al., 2023; Xu et al., 2023) and numerous computer techniques have been created to anticipate DNA 4mC sites. Both standard machine learning techniques and more current deep learning algorithms have been used to provide a strong result. In the field of 4mC site prediction, researchers have made significant strides by leveraging machine learning algorithms. These approaches utilize computational models to identify and classify 4mC sites within DNA sequences. Various machine learning techniques have been explored, including support vector machine (SVM) (Chen et al., 2017), random forest (RF), Markov model (MM), and ensemble methods. Additionally, advanced techniques such as extreme gradient boosting (XGBoost) and Laplacian Regularized Sparse Representation have also been employed in this context (Chen et al., 2017; Manavalan et al., 2019; He et al., 2019; Hasan et al., 2020; Zhao et al., 2020; Ao et al., 2022b; Xiao et al., 2022). However, traditional machine learning algorithms rely significantly on data representations known as features for appropriate performance, and it's tough to figure out which features are best for a certain task. Deep learning overcomes the limitations of traditional methods by offering adaptivity, fault tolerance, nonlinearity, and improved input-to-output mapping. Deep learning methods, such as convolutional neural networks (CNNs) and recurrent neural networks (RNNs), have been developed for the detection of 4mC sites, leveraging their ability to capture sequence patterns and dependencies, thereby contributing to accurate identification of these sites and enhancing our understanding of DNA methylation in gene regulation and epigenetics (Xu et al., 2021; Liu et al., 2022). Yet there are still many deep learning methods that have not been applied, which have achieved great success in various application scenarios, including computer vision, speech recognition, biomarker identification (Zeng et al., 2020; Cai et al., 2021) and drug discovery (Chen et al., 2021; Zhang et al., 2021; Hu et al., 2022b; Dong et al., 2022; Pan et al., 2022; Song et al., 2022).

Choosing an appropriate deep learning model for bioinformatics problems poses a significant challenge for biologists. Understanding and comparing the performance of different models on specific datasets is of paramount importance for guiding practical applications. Therefore, our research focuses on evaluating the performance of multiple deep learning models on the 4mC datasets, aiming to assist biologists in making informed decisions when selecting suitable models.

We selected several common deep learning models, including RNN (Recurrent Nerual Network) (Rumelhart et al., 1986), long short-term memory (LSTM) (Graves, 2012), bi-directional long short-term memory (Bi-LSTM) (Graves and Schmidhuber, 2005; Sharma and Srivastava, 2021), text convolutional neural network (Text-CNN) (Kim, 2014), and bidirectional encoder representations from transformers (BERT) (Ji et al., 2021; Tran and Nguyen, 2022), and compared their performances on the 4mC datasets through optimization of model hyperparameters. Our research findings provide strong evidence-based support for biologists, aiding them in making informed choices when addressing bioinformatics problems on the 4mC datasets. By comparing the performance of multiple models, we can offer recommendations tailored to different problems and datasets, enabling biologists to better understand and leverage the advantages of deep learning models.

## Materials and methods

**FIGURE 1:**
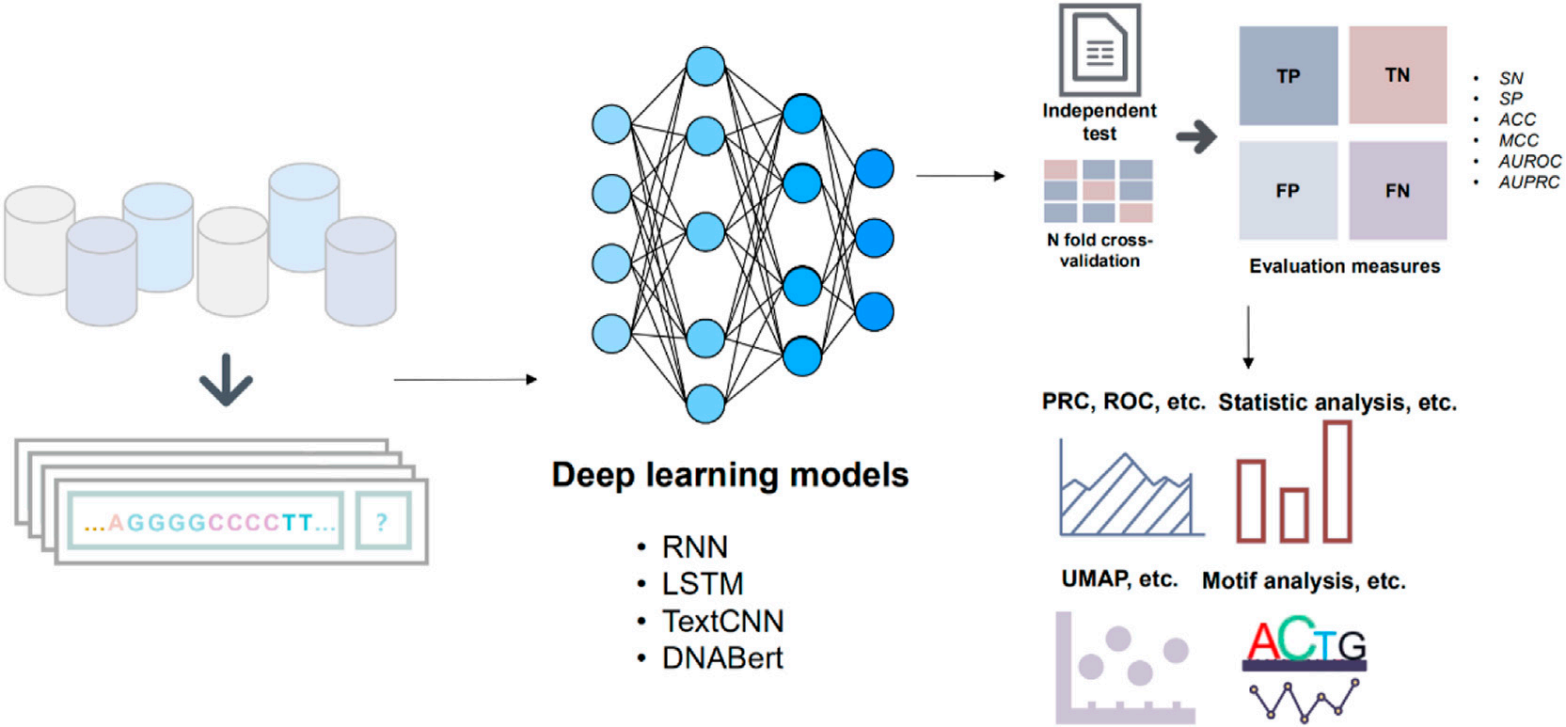
The workflow of the main modules. The benchmark datasets are initially divided into training and test sets. Subsequently, the divided dataset is fed into various deep learning models for prediction. The results of the predictions from different models are then evaluated. Finally, the data generated throughout these steps are visualized and analyzed for further insights.

The implementation of our experiments relies on the DeepBIO (Wang et al., 2022) platform, which provides a wide selection of deep learning models and a visual comparison of multiple models. Figure 1 illustrates the overall framework of our works. We selected four deep learning models (RNN, LSTM, Bi-LSTM, Text-CNN) and pre-trained BERT models from the DeepBIO platform, and BERT is used as our main method to compare with other methods.

## Datasets

The first step in creating a strong and trustworthy classification model is creating high-quality benchmark datasets. In this study, six benchmark datasets were utilized (Yu et al., 2021). Table 1 provides a statistical summary of the datasets. The positive samples consisted of sequences that were 41 base pairs (bp) in length and contained a 4mC (4-methylcytosine) site located in the middle. These datasets have undergone rigorous preprocessing and quality control measures to ensure data accuracy and consistency (Jin et al., 2022). By training and evaluating the model on data from multiple species, including humans, animals, and plants, we ensure its broad applicability and provide valuable insights for biologists in selecting deep learning models.

**TABLE 1:** Statistical summary of benchmark datasets.

Species	Positive sample	Negative sample	Total
*C. elegans*	1,554	1,554	3,108
*D. melanogaster*	1,769	1,769	3,538
*A. thaliana*	1,978	1,978	3,956
*E. coli*	388	388	776
*G. subterraneus*	906	906	1,812
*G. pickeringii*	569	569	1,138

## Input feature matrix

Deep learning algorithms possess the capability to autonomously extract valuable features from data, distinguishing them from conventional machine learning methods that necessitate manual feature engineering. Nonetheless, when dealing with a string of nucleotide letters (A, C, G, and T), a conversion into a matrix format is required prior to feeding it into a neural network layer. Unlike prior methods that used several feature encodings schemes to represent the sequence as the input to train the model, this method uses a single feature encoding scheme. We took the dictionary encoding approaches for representing DNA sequences. To represent DNA sequences, we utilized a dictionary encoding method where each nucleotide (A, C, G, and T) is assigned a numeric value. Specifically, A is represented by 1, C by 2, G by 3, and T by 4. This encoding scheme allows us to convert the sequence into an N-dimensional vector, facilitating its input into the neural network for further analysis.

## Model construction and parameters

We have selected deep-learning models that have received a lot of attention in recent years as follows: RNN, LSTM, Bi-LSTM, Text-CNN, and BERT. The first four deep learning models we used are the models provided by the DeepBIO platform with parameters already set and the BERT model we used is pre-trained DNABERT (Ji et al., 2021; Ren et al., 2022), which achieves the best performance on several DNA sequence classification tasks.

RNN is a type of neural network where the output of the previous neuron is fed back as input to the current neuron, creating temporal memory and enabling the processing of dynamic input sequences. RNNs find wide applications in various domains, including voice recognition, time series analysis, DNA sequences, and sequential data processing. One notable variant of RNNs that addresses the issue of capturing long-term dependencies is Long Short-Term Memory (LSTM). LSTM introduces a cell state that serves as a memory component, allowing the network to retain relevant information over extended periods. The forget gate in LSTM controls which information should be discarded and retained by using a sigmoid activation function. Additionally, Bidirectional LSTM (BiLSTM) processes input data in both forward and backward directions, effectively incorporating information from both past and future states. This bidirectional approach enables BiLSTM to capture intricate sequential relationships between words and sentences, making it particularly advantageous for Natural Language Processing (NLP) tasks that require contextual information from both preceding and succeeding elements in the input sequence. The RNN, LSTM, and Bi-LSTM architectures consist of stacked RNN cells, LSTM cells, and bidirectional LSTM cells, respectively. All these architectures share a similar structure, featuring 128 hidden neurons and a single layer for optimal performance. To prevent overfitting and promote generalization, a dropout rate of 0.2 was applied, and the output layer utilized sigmoid activation with a single neuron.

Text-CNN, a powerful deep learning approach for language classification tasks, such as sentiment analysis and question categorization, is a convolutional neural network tailored for text processing. The core structure comprises four layers: an embedding layer, a convolution layer, a pooling layer, and a fully connected layer. In our implementation, we set four convolutional kernel sizes (1, 2, 4, 8), and the number of convolutional kernels is uniformly set to 128. The embeddings undergo convolutional operations with a sliding kernel, producing convolutions that are subsequently downsampled through a Max Pooling layer to manage complexity and computational requirements. The scalar pooling outputs are then concatenated to form a vector representation of the input sequence. To mitigate overfitting, regularization methods, including a dropout layer with a rate of 0.2 and ReLU activation, are employed in the penultimate layer, preventing overfitting of the hidden layer.

BERT, an abbreviation for Bidirectional Encoder Representations from Transformers, originates from the Transformer architecture. In the Transformer model, every output element is intricately connected to every input element, with dynamically calculated weightings based on their connections. BERT is a pre-trained model that benefits from its ability to learn rich contextualized representations by considering the entire input sequence during training. Our study employs the pre-trained DNABert model, which has demonstrated superior performance in several DNA sequence classification tasks. We specifically fine-tune the 6mer-BERT variant on the 4mC methylation site benchmark dataset. Fine-tuning a pre-trained model on a task-specific dataset allows us to transfer the knowledge acquired during pre-training, enabling the model to achieve state-of-the-art performance in predicting DNA 4mC methylation sites. The incorporation of BERT’s pre-trained knowledge provides significant advantages, as the model has already learned from vast amounts of data and captures intricate sequence patterns and dependencies. By leveraging pre-trained models like BERT, we achieve robust and accurate predictions, even in scenarios with limited training data.

## Evaluation metrics

In order to compare with previous related work, we selected the commonly used evaluation indicators comprised of accuracy (ACC), sensitivity (SN), specificity (SP), Matthews’ coefficient correlation (MCC), and area under the receiver operating characteristic curve (AUC). These indicators are calculated by the following formula:

$$\left\{\begin{array}{c} \begin{array}{c} ACC=\frac{TP+TN}{TP+TN+FP+FN}\\ Sensitivity=\frac{TP}{TP+FN}\\ Specificity=\frac{TN}{TN+FP} \end{array}\\ MCC=\frac{TP\times TN-FP\times FN}{\sqrt{\left(TP+FN\right)\left(TP+FP\right)\left(TN+FP\right)\left(TN+FN\right)}} \end{array}\right.$$

where *TP* represents true positives, which is the number of correctly predicted positive samples; *TN* represents true negatives, which is the number of correctly predicted negative samples; *FP* represents false positives, which is the number of negative samples wrongly predicted as positive; and *FN* represents false negatives, which is the number of positive samples wrongly predicted as negative.

## Experimental setup

In our experimental design, we adopted the default settings of DeepBIO for other hyperparameters. For instance, when performing data set deduplication, we limited the duplication rate to 0.8 using the CDHIT algorithm integrated in the DeepBIO platform. Furthermore, we conducted a grid search on hyperparameters such as learning rate and batch size for each model. Grid search is a method for hyperparameter tuning, where different combinations of hyperparameters are tried to determine the optimal model configuration. Such experimental settings ensure that the models achieve their maximum potential performance while maintaining the reliability, fairness, and accuracy of the experiments.

## Result

In this section, we evaluate the performance of the different models and analyze the features extracted by the different models. In addition, we also compare the features learnt from deep-learning models with the traditional manual feature extraction methods applied in other studies to further demonstrate the superiority of deep learning in solving the 4mC methylation site detection problem. To ensure a balanced representation, the samples were randomly divided into training and test datasets for each species. The division was done in a ratio of 9:1, with 90% of the samples allocated to the training dataset and the remaining 10% assigned to the test dataset.

### Performance evaluation of multiple models

We conducted a comprehensive performance evaluation of four different models on six datasets to assess their performance in various data environments. The evaluation process involved the use of common binary classification metrics, such as accuracy (ACC), sensitivity, specificity, area under the curve (AUC), and Matthews correlation coefficient (MCC), to provide a comprehensive understanding of the models’ classification capabilities and highlight their performance differences. In addition to these metrics, we also employed receiver operating characteristic (ROC) curves and precision-recall curves (PRC) to further analyze the models’ performance.

**FIGURE 2:**
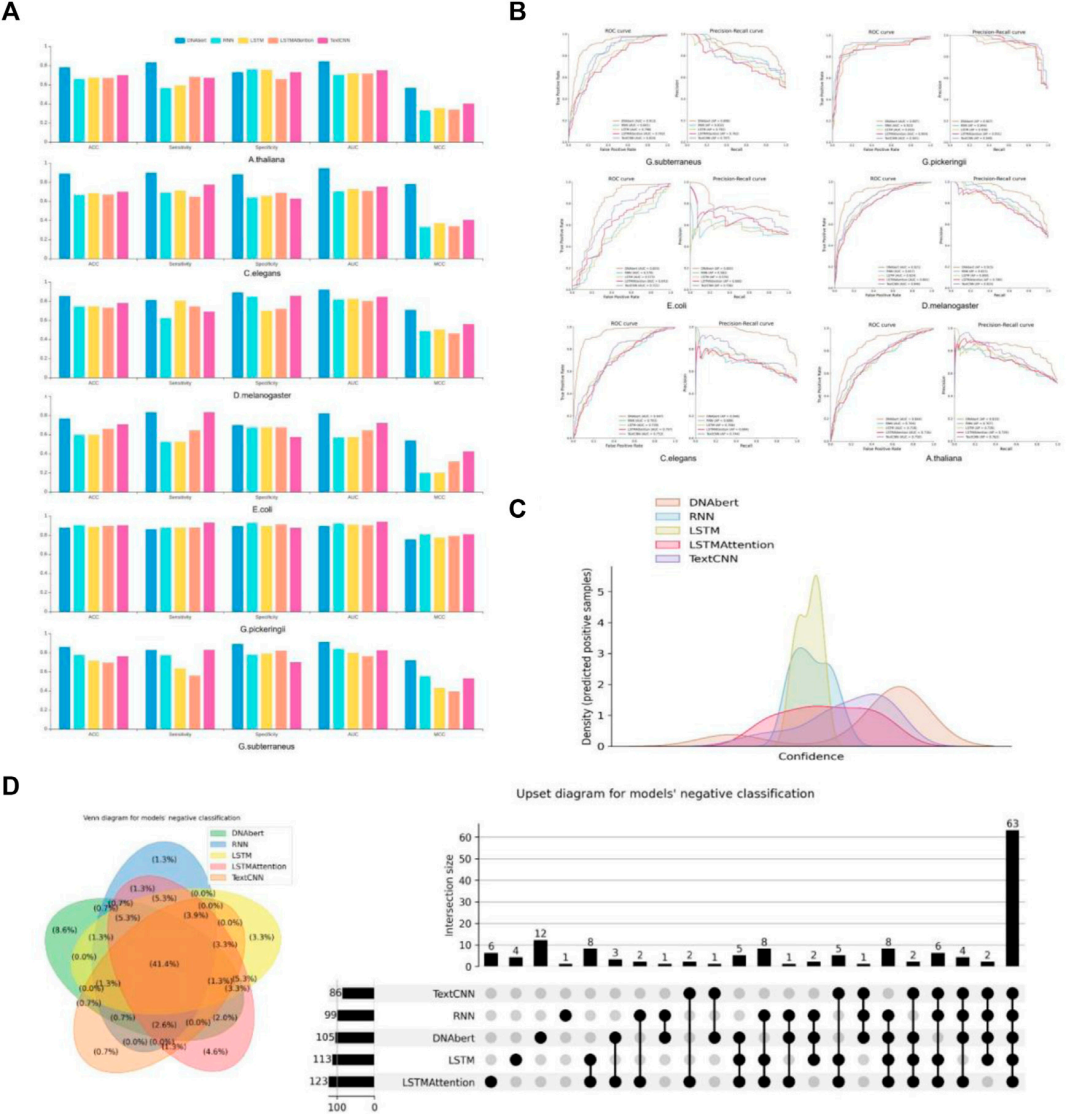
Performance evaluation of multiple models. **(A)** The basic statistics of ACC, Sensitivity, Specificity, AUC, and MCC in different models. **(B)** Performance comparison between DNABERT and other state-of-the-art methods on the benchmark datasets. **(C)** Density distribution of the prediction confidence by different deep learning models. **(D)** VENN and Upset plots show the overlap of different models’ predictions.

Throughout our evaluation, we observed variations in performance across different datasets. While certain models demonstrated superior predictive performance on most datasets, their performance might vary on specific datasets. As shown in Figures 2A, B, the RNN and TextCNN models exhibited promising performance on the *G. pickeringii* dataset, while DNABERT outperformed others on the *G. subterraneus* dataset. Overall, DNABERT consistently showcased superior performance across the evaluated datasets.

Furthermore, let’s consider the results obtained on the *E. coli* dataset. The density distribution of prediction confidence by different deep learning models (Figure 2C) provides insights into the prediction preferences of each model. In the case of LSTM and Text-CNN, their density distribution shows a preference towards the center part of the X-axis, around 0.5. This indicates their poor binary classification ability and confusion in distinguishing between positive and negative instances. On the other hand, the density distribution for DNABERT is skewed towards the right side of the X-axis, indicating a better classification performance. This suggests that DNABERT exhibits a stronger ability to differentiate between positive and negative instances. And this is consistent with the conclusions drawn from the performance comparison in Figure 2A.

We also performed statistics on the overlap of predictions between different models for the same dataset. Take the results obtained on the *G. subterraneus* dataset as an example, the distribution of sets classified as negative classes by different models in the test set is shown in Figure 2D. In the VN diagram on the left, 41.4% of the test set is judged as negative by all models (negative classes account for 50% of the test set in total). The difference in quantity is shown more clearly in the right figure, and we can find that DNABERT may be one of the less effective models for classification under this dataset, as it predicts more negative cases individually. However, given that most of the model predictions converge on the same, we can conclude that most of the models are consistent in their classification results.

### Deep learning model feature analysis

**FIGURE 3:**
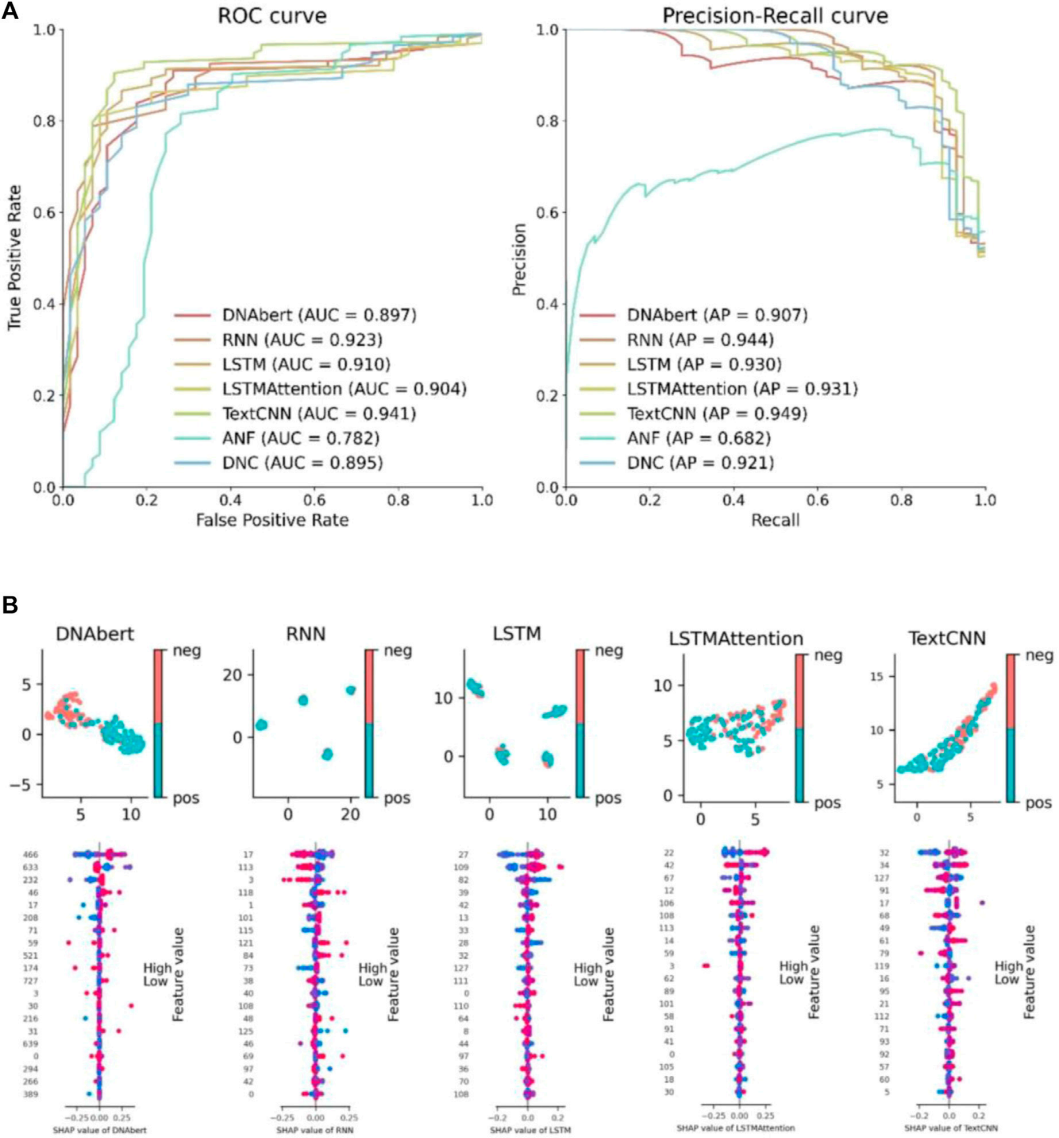
Deep learning model feature analysis. **(A)** Feature performance comparison between hand-crafted features and the features learned by deep learning models. **(B)** UMAP feature visualization and SHAP feature importance visualization.

We conduct a comparative study on the features learned by deep learning from biological information. This includes comparisons between different deep learning models as well as comparisons between deep learning features and manually designed features. By conducting feature comparisons, we aim to further validate the superiority of deep learning methods and enhance the interpretability of deep learning models. We select ANF, binary, CKSNAP, and DNC approaches to extracting features and using SVM for unsupervised classification to compare with our deep learning models. Figure 3A presents the ROC and PR curves for all models on the *G. pickeringii* dataset. We only display the two best-performing traditional manual feature methods for comparison. It is evident that most of the deep learning methods outperform the traditional approaches in terms of classification performance.

To visualize the results of deep learning features, we utilized UMAP (Uniform Manifold Approximation and Projection) and SHAP (Shapley Additive Explanations) plots for display (Figure 3B). The UMAP plot reduces the dimensionality of the features while preserving the underlying data structure. It enables data clustering and categorization by mapping high-dimensional features into a lower-dimensional space, allowing for an analysis of feature similarity between positive and negative instances. The SHAP plot facilitates the understanding of feature importance and contribution to model predictions, providing interpretability to the model and enabling comparison of feature impacts. It helps to comprehend the significance of features in model predictions, enhancing interpretability and facilitating comparison among different features. In the feature visualization figure, each row corresponds to a specific feature, and the x-axis represents the snap value, providing a clearer understanding of the feature. The color gradient indicates the feature value, with higher values represented by redder colors and lower values represented by bluer colors. Each line represents a feature, and the horizontal position represents the SHAP value assigned to that feature in a particular sample. Each point represents a sample. The intensity of the color reflects the impact of the feature, with redder colors indicating a larger impact and bluer colors indicating a smaller impact. The scattered distribution of points indicates a greater influence of the feature.

## Conclusion

In this study, we use several currently popular deep learning models on the problem of 4mC methylation detection of DNA. We first present the current status of DNA 4mC methylation site detection, followed by the design of deep learning model workflows on six benchmark datasets, and finally, we evaluate the output of all models and conclude that deep learning has great potential for methylation detection, leading the way to future sequencing technologies along with newer bio-experimental methods. In fact, deep learning methods consistently outperformed traditional machine learning methods on all datasets. Furthermore, it was observed that pre-trained deep learning models with a higher number of parameters exhibited even better performance. We believe this may be because deep learning models with more parameters capture more features and analyze the features acquired by each model. By attempting to explain the model’s internal workings and shed light on its internal representations, we aim to define its “black box” behavior.

## Data Availability

The original contributions presented in the study are included in the article/Supplementary Material, further inquiries can be directed to the corresponding authors.
